# Thoracobifemoral bypass for radiation-induced paravisceral aortic stenosis

**DOI:** 10.1016/j.jvscit.2025.101883

**Published:** 2025-06-17

**Authors:** Nicola M. Habash, Calvin L. Chao, Nidhi K. Reddy, Nicholas Lysak, Mark K. Eskandari

**Affiliations:** Division of Vascular Surgery, Department of Surgery, Northwestern University Feinberg School of Medicine, Chicago, IL

**Keywords:** Thoracobifemoral bypass, Aortobifemoral bypass, Extra-anatomical revascularization, Radiation-induced arteriopathy, Abdominal aortic occlusive disease

## Abstract

Radiation-induced arterial disease is a delayed complication of childhood cancer therapy. We present the case of a 56-year-old man with disabling claudication secondary to severe paravisceral aortic stenosis, attributed to abdominal radiation for Wilms tumor in early childhood. Extensive periaortic fibrosis and calcification precluded endovascular repair and conventional aortobifemoral bypass. He underwent successful thoracobifemoral bypass via a left anterolateral thoracotomy approach and retroperitoneal tunneling of the graft. This case highlights the challenges of managing radiation-associated vascular disease. In select patients, thoracobifemoral bypass offers a durable solution when traditional approaches are contraindicated owing to hostile intraabdominal anatomy.

Radiation-induced arterial disease is a delayed but recognized complication of therapeutic radiation and is increasingly encountered as cancer survivorship improves.^1^ Chronic radiation injury accelerates atherosclerosis and can lead to vasculature fibrosis, stenosis, thrombosis, or aneurysm formation.^1^ The management of radiation-induced arterial stenosis is challenging owing to poor tissue quality, scarring, and impaired healing in irradiated fields.^2^ Additionally, circumferential calcification, dense mural fibrosis, and inflammatory vasculopathy of the aorta may preclude endovascular revascularization.

Although open aortobifemoral bypass (ABF) is the standard approach for chronic atherosclerotic abdominal aortic occlusion,^3^ radiation-altered abdominal fields may render a transabdominal approach hazardous and prohibitively risky.^4^ This report describes a successful thoracobifemoral bypass (TBF) for paravisceral aortic stenosis in a Wilms tumor survivor, highlighting the operative strategy. The patient provided consent for case publication.

## Case report

### History

A 56-year-old man with coronary artery disease, hypertension, hyperlipidemia, type 2 diabetes mellitus, a smoking history, and childhood Wilms tumor (left nephrectomy and abdominal radiation therapy at age 3) presented with short-distance claudication. He reported disabling claudication at approximately 50 feet. He denied resting pain, abdominal pain after meals, or weight loss. On examination, bilateral femoral and distal pulses were diminished, whereas upper extremity pulses were normal. There were no focal neurological deficits.

**Fig 1:**
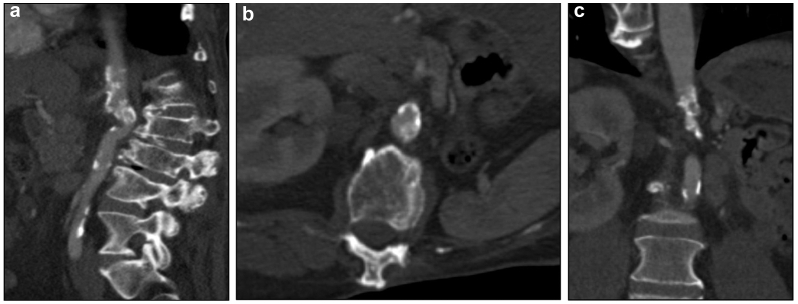
Preoperative computed tomography angiography (CTA) demonstrating high-grade aortic stenosis in the **(a)** sagittal, **(b)** axial, and **(c)** coronal planes.

Arterial duplex ultrasound examination showed monophasic, low-resistance waveforms with a prolonged upstroke beginning at the common femoral arteries (CFA), consistent with hemodynamically significant aortoiliac inflow disease. The ankle-brachial index (ABI) was 0.66 on the right and 0.64 on the left. Computed tomography angiography (CTA) demonstrated severe paravisceral aortic stenosis with near occlusion just below the superior mesenteric artery (SMA) and single renal artery (Fig 1). The narrowing was surrounded by dense periaortic fibrosis and calcification. The origins of the SMA and celiac artery had moderate calcified plaque without high-grade stenosis. The right renal artery was narrowed mildly. Both common iliac arteries had moderately calcified plaque without flow-limiting stenosis. Bilateral CFAs were free of disease. No evidence of active malignancy or intra-abdominal pathology was identified. The findings were consistent with radiation-induced arteriopathy. After failure to alleviate his symptoms with several months of structured exercise therapy, we elected to restore perfusion to the lower extremities with a TBF.

### Description of the procedure

**Fig 2:**
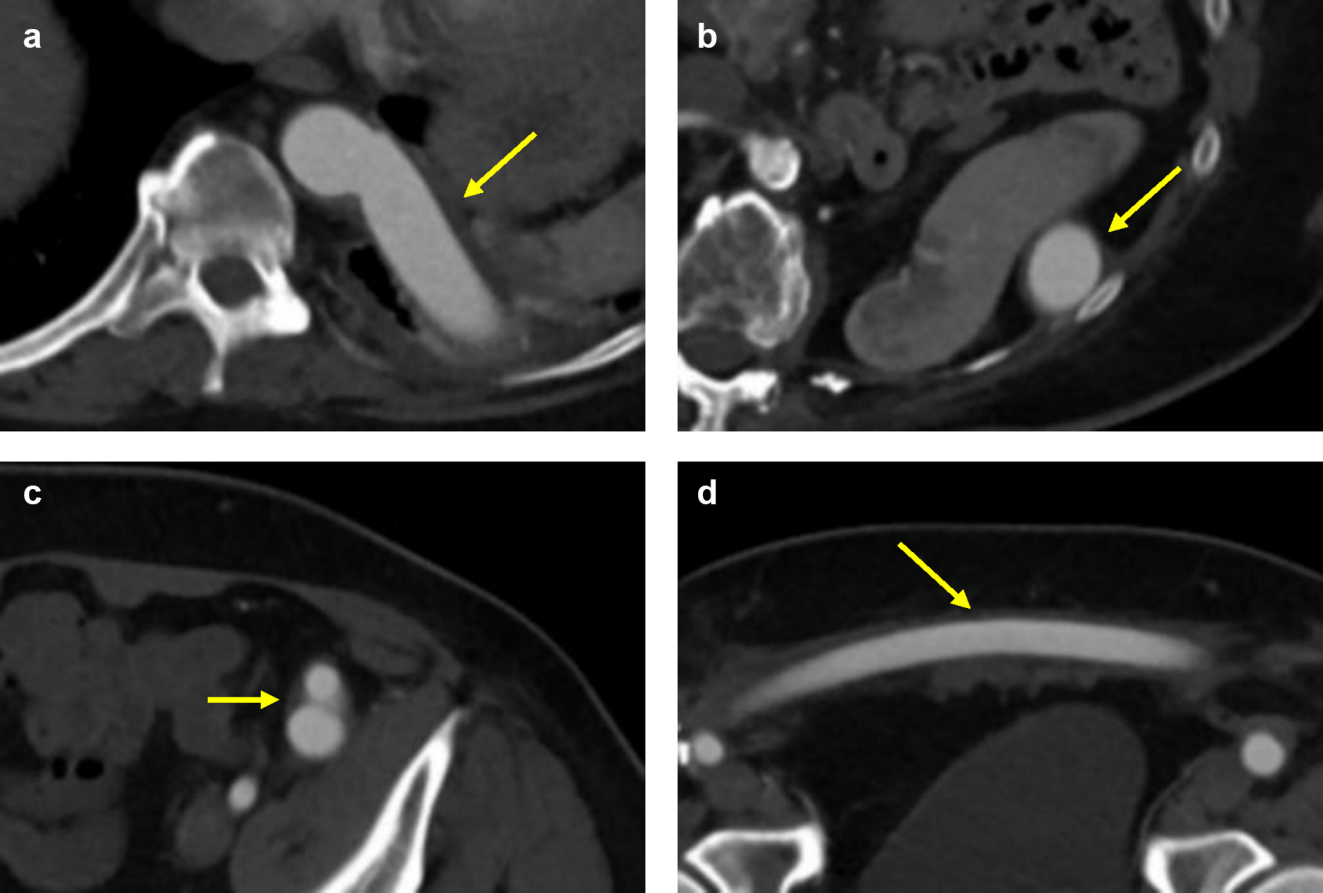
Axial computed tomography angiography (CTA) demonstrating the graft (yellow arrow) as it traverses the diaphragm and enters the retroperitoneal space **(a)**, passes deep to the spleen **(b)**, reaches the level of graft bifurcation **(c)**, and courses subcutaneously toward the right groin **(d)**.

The patient was placed in the right lateral decubitus position. Bilateral groin incisions were made, exposing the CFA, superficial femoral artery, and the profunda femoris arteries. On the left side, the epigastric vein was doubly ligated and divided. The inguinal ligament was mobilized fully to create a pocket for the bifurcated portion of the graft, and dissection was extended into the mid-external iliac artery, which was free of adhesions. A left anterolateral thoracotomy was performed through the seventh intercostal space. The left lung was deflated with a bronchial blocker, the inferior pulmonary ligament was divided, and the decompressed lung was packed into the superior aspect of the chest cavity. The diaphragm was retracted caudad, and the thoracic aorta was exposed with preservation of the intercostal arteries. A retroperitoneal tunnel was fashioned from the left groin in the anterior lateral space, up through the diaphragm, and above the spleen with blunt dissection. The descending thoracic aorta was mobilized freely. After systemic heparinization, intravenous mannitol, and furosemide administration, the aorta was clamped partially with a side-biting clamp at T10. A longitudinal arteriotomy was performed, and an 18-mm Dacron tube graft, previously sewn to an 18-mm bifurcated graft, was sewn end-to-side to the descending thoracic aorta using a running 3-0 Prolene suture. The total clamp time was approximately 25 minutes. The graft, consisting of a tube-to-bifurcated configuration, was tunneled down in the anterior retroperitoneal space (Fig 2) with the bifurcation positioned just above the level of the left inguinal ligament. The right graft limb was tunneled subcutaneously to the right groin. End-to-side anastomoses were performed to the left and right CFAs with running 5-0 Prolene suture. Proximal and distal pulses were reestablished bilaterally. To minimize direct contact between the lung and the graft, a pleural flap was fashioned from the lateral chest wall pleura and sutured over the proximal anastomosis, potentially reducing the risk of graft erosion. No specific measures were taken to reduce the risk of pleural adhesions. The graft was then draped over the diaphragm into the retroperitoneal tunnel and a 28F chest tube was placed. The lung was reexpanded, and the thoracotomy was closed in layers. The postoperative ABI improved to 0.95 on the right and 0.90 on the left.

### Postoperative course and follow-up

The patient was extubated immediately postoperatively. He developed transient acute kidney injury (peak creatinine, 1.5 mg/dL) that resolved with intravenous fluids. He remained off oral feeding until postoperative (POD) 1, after which he tolerated oral intake. A stable apical pneumothorax was noted after chest tube removal on POD 4, and he required supplemental oxygen and diuresis for pulmonary edema. He was discharged home on POD 9.

**Fig 3:**
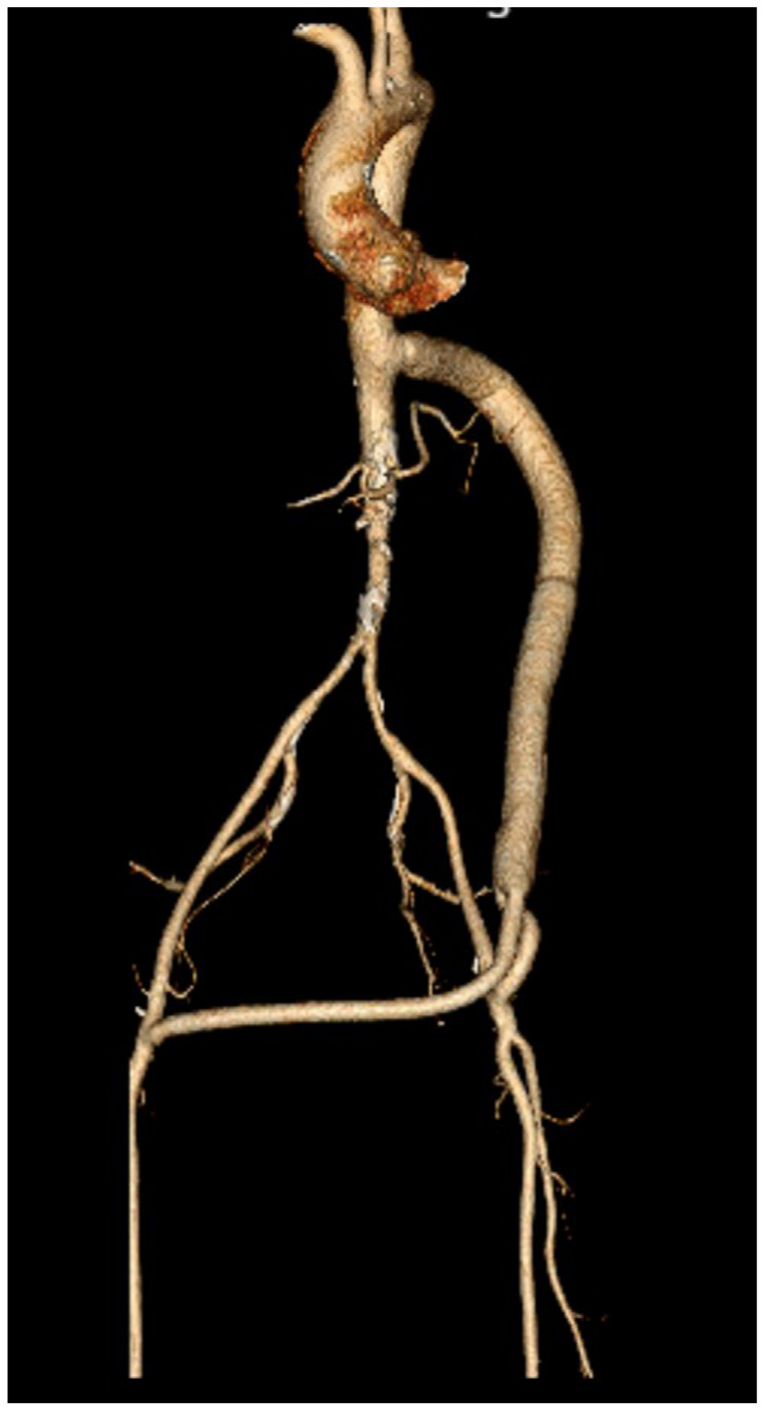
Three-dimensional computed tomography reconstruction showing a thoracobifemoral bypass (TBF) graft originating from the descending thoracic aorta and extending to the bilateral common femoral arteries (CFAs).

At 6 months, he reported complete resolution of claudication and had resumed full activity. Bilateral femoral, popliteal, and pedal pulses were strong. The ABI was 1.04 on the right and 0.95 on the left. Duplex ultrasound examination demonstrated triphasic waveforms in both superficial femoral arteries. CTA demonstrated a widely patent TBF bypass without evidence of a pseudoaneurysm or leak (Fig 3). At 18 months, he remained asymptomatic without graft complications. The ABI was 1.11 on the right and 1.01 on the left. The 2-year postoperative surveillance CTA redemonstrated a patent bypass.

## Discussion

Vascular reconstruction in previously irradiated fields poses unique difficulties. This case illustrates the phenomenon of radiation-induced large artery stenosis and highlight considerations in its management.

Endovascular repair with an aortic stent graft may be considered to restore paravisceral and infrarenal aortic patency without the morbidity of open surgery for typical atherosclerotic disease. Additionally, endovascular treatment in radiation-damaged large arteries can be attempted and may be successful in select cases. However, the stenotic segment in our case was long and adjacent to the origins of the visceral branches, making endovascular repair complex, even with advanced fenestrated techniques. Balloon angioplasty and stenting might risk aortic rupture or coverage of visceral arteries. Ultimately, we judged that an endovascular approach carried high risk and uncertain durability.

ABF was not a viable option owing to the absence of healthy infrarenal aortic inflow sites. In patients with severe visceral aortic stenosis, ABF is generally discouraged, because constructing an infrarenal anastomosis is often not feasible.^4^ Furthermore, it is recommended that bypass targets lie outside the radiation-exposed field.^5^ An open in situ repair with aortic endarterectomy was also considered. However, abdominal exposure would likely have necessitated bypasses to the right renal artery and SMA, with the possible use of a synthetic graft if the lesion could not be everted entirely. Operative entry into the previously irradiated abdomen posed significant risks. Tissue planes were likely obliterated by fibrosis and prior surgery, and important structures such as the ureters could be adherent, increasing the risk of injury. Additionally, radiation-injured tissues exhibit impaired healing and a heightened susceptibility to infection.

In a previously reported case involving an irradiated pelvis after Ewing sarcoma in childhood, the authors described severe fibrosis and limited working space, rendering aortoiliac exposure and in-line aortic reconstruction difficult.^6^ Consequently, it is recommended to use an extra-anatomical bypass to avoid the radiation-compromised areas.^5^ Similarly, we selected an extra-anatomical route to avoid the hostile field and achieve durable inflow from a healthy proximal source. Options typically include ABF bypass or TBF bypass. The former is known to have a lower long-term patency.^7^ Although TBF bypass is associated with high perioperative complication rates and should be reserved for cases with compelling anatomical indications, such as radiation injury,^8^ it remains a valuable option when in-line reconstruction is not feasible. Minimal thoracotomy TBF bypass has also been proposed as an alternative with a reduction in thoracotomy size at the price of a flank counter incision.^9^ This approach uses a second cardiac surgery team and potentially limits operative time, although it is not used routinely at our institution.

## Conclusions

Radiation-induced large artery stenosis can result decades after chemoradiation for intra-abdominal malignancy. This case underscores the importance of long-term vascular follow-up in patients who received radiation therapy in childhood. Our patient's case demonstrates that, with careful planning, an extra-anatomical TBF bypass can restore blood flow with excellent results when other options are limited. Key considerations include selecting a healthy inflow site, avoiding diseased and fibrotic areas, and anticipating the technical difficulties associated with radiation damage. For the growing population of cancer survivors, a heightened awareness of radiation-associated vascular disease is warranted. This case also reinforces that, even in the endovascular era, mastery of open aortic bypass techniques remains essential.

## Funding

None.

## Disclosures

M.K.E. is a paid consultant with W. L. Gore & Associates and Boston Scientific (Silkroad Medical).
